# Incidence of Vitamin D Deficiency and Its Association With Microalbuminuria in Patients With Type 2 Diabetes Mellitus

**DOI:** 10.7759/cureus.45854

**Published:** 2023-09-24

**Authors:** Muhammad Hamza Riaz, Ammar Jamil, Hira Yousaf, Muhammad Hassan, Muhammad Ahmer Sohaib, Sharjeel Babar, Muhammad Hassan Ahmad, Ibtesam Allahi, Muhammad Zeshan Mehmood, Tayyab Mumtaz Khan

**Affiliations:** 1 Cardiology, Allama Iqbal Medical College, Lahore, PAK; 2 Emergency Medicine, Holy Family Hospital, Rawalpindi, PAK; 3 Analytical Chemistry, University of Huddersfield, Huddersfield, GBR; 4 Internal Medicine, Allama Iqbal Medical College, Lahore, PAK; 5 Medicine, Allama Iqbal Medical College, Lahore, PAK; 6 Internal Medicine, Mayo Hospital, Lahore, PAK; 7 General Surgery, Allama Iqbal Medical College, Lahore, PAK; 8 Surgery, Benazir Bhutto Hospital, Rawalpindi, PAK; 9 Surgery, Rawalpindi Medical University, Rawalpindi, PAK

**Keywords:** diabetes mellitus, type 2, patients, microalbuminuria, association, deficiency, vitamin d, incidence

## Abstract

Background

Vitamin D (VD) deficiency is common in patients with type 2 diabetes mellitus (T2DM). VD deficiency and its associated factors are understudied in Pakistan. This study aimed to estimate the incidence of VD deficiency and its association with microalbuminuria in patients with T2DM.

Methods

This descriptive cross-sectional study was performed on 110 patients diagnosed with T2DM aged between 30 and 65 years in the outpatient department clinic of diabetes in Benazir Bhutto Hospital, Rawalpindi, for around eight months from November 2022 to June 2023. Non-probability sampling technique and established inclusion and exclusion criteria were used for patient recruitment. Ethical approval and informed consent were also waived before data collection. Data collection was done by an interview-based and self-designed questionnaire. Data analysis was carried out via descriptive statistics along with chi-square, independent-samples t-test, and Pearson correlation in Statistical Package for the Social Sciences (SPSS) version 25 (Armonk, NY: IBM Corp.).

Results

The means of the study population for age, serum VD, and UACR (urine spot for albumin-to-creatinine ratio) were 48.50±15.67 years, 28.16±15.34 ng/mL, and 29.69± 87.22 µg/mg, respectively. The incidences of VD deficiency and microalbuminuria in the study population were 43.64% and 28.20%, respectively. VD deficiency was significantly associated with age group (p=0.002), gender (p=0.008), and albuminuria status (p=0.004). The comparison of means of UACR between the VD deficiency group and the non-VD deficiency group was also significant (0.001). VD deficiency was higher among older age groups, female gender, and patients with microalbuminuria. A significant negative correlation between serum VD level and UACR (microalbuminuria) (p=0.002) was present.

Conclusion

VD deficiency incidence was notably high in the study population. Older age, female gender, and microalbuminuria were found to elevate the VD deficiency to a crucial level. Serum VD deficiency and microalbuminuria were significantly and negatively correlated. Therefore, VD level should be monitored intermittently in T2DM, so that we could prevent the progression of T2DM timely.

## Introduction

Type 2 diabetes mellitus (T2DM) is a global public health problem and it is characterized by insulin deficiency or insulin resistance in the body cells, which leads to raised blood glucose levels [1]. In 2019, approximately 463 million people had diabetes mellitus and it is also estimated in 2019 that this number would rise to 700 million by the end of 2045 [2]. A huge number of the Pakistani population is suffering from diabetes mellitus. In accordance with the WHO (World Health Organization) data, nearly 10% (12.9 million) of the Pakistani population has diabetes mellitus [3].

T2DM has many chronic complications including microvascular and macrovascular. These complications bring a decline in the quality of life and raise the load on healthcare systems and diabetes-linked mortality. Every year in Pakistan, T2DM because of its associated complications almost leads to 120,000 deaths [1,3]. Therefore, it is necessary to recognize the modifiable factors associated with the progression of these complications, and by working on them we could bring improvement in the prognosis of T2DM.

Vitamin D (VD) is an important hormone that plays a role in the pathogenesis of T2DM by its action on pancreatic B cells and regulation of insulin resistance in body cells [4]. In previous human studies, it has been shown that VD deficiency leads to impaired glucose tolerance while supplementation of VD along with calcium brings improvement in the metabolism of glucose [5,6]. The prevalence of proteinuria, which is an indicator of renal involvement in T2DM patients, goes up with the decrease in VD level [7]. VD contributes to suppressing renin production while its VD deficiency causes renal injury progression in diabetic patients [4,8].

Although association between the VD deficiency and T2DM complications, especially with diabetic nephropathy, has been studied in different parts of the world [4,6,9], VD deficiency and its association with microalbuminuria is understudied in Pakistan. Therefore, the present study aimed to determine the incidence of VD deficiency and its association with microalbuminuria in patients diagnosed with T2DM.

## Materials and methods

Study design and study population

We conducted this single-center cross-sectional study in the outpatient department (OPD) clinic of diabetes in Benazir Bhutto Hospital, Rawalpindi, on 110 patients, for almost eight months from November 2022 to June 2023.

Inclusion and exclusion criteria

Established inclusion and exclusion criteria along with the non-probability convenient sampling technique were utilized for the recruitment of the study participants. All patients with age less than 30 years or above 65 years, and patients who had other types of diabetes like diabetes mellitus type 1, gestational diabetes, chronic kidney disease, acute infection, liver disease, bowel disease-related malabsorption, hyperparathyroidism, and hypoparathyroidism were excluded from the study. We also excluded patients who were taking any drugs (steroids, anticonvulsants, cholestyramine, rifampin, and even VD and calcium supplements) that affect VD level and patients with macroalbuminuria (urine spot for albumin-to-creatinine ratio [UACR] above 300 µg/mg), whereas only those patients whose age range was between 30 and 65 years with T2DM and who did not fulfill the above exclusion criteria of this study were included into the current study.

Ethics

Ethical Review Board (ERB) of Benazir Bhutto Hospital, Rawalpindi, granted ethical approval for the current study. The ethical approval number for this study is BBH.ERB.283/205. After elaborating the objectives of the study to the research population, informed consent was also obtained from each participant of the study.

Data collection

Data collection was carried out via self-designed proforma. It had two parts. Demographic data of the study population like gender (male or female) and age group (30 to 45 years or above 45 to 65 years) was noted in the first part of the proforma, while clinical parameters of the study population such as serum VD levels and albuminuria status were recorded in the second part of the proforma. The patients were divided into two groups according to their VD levels. Patients with VD levels less than 20 ng/mL were labeled as the VD deficiency group while patients with VD levels between 20 and 30 ng/mL (VD insufficiency) and patients with VD levels above 30-50 ng/mL (vitamin sufficiency/optimum level) were labeled as non-VD deficiency group. Serum VD level quantification was carried out via high-performance liquid chromatography. Albuminuria status was determined in midstream urine samples. Patients with UACR less than 30 µg/mg were considered to have normo-albuminuria whereas patients with UACR between 30 and 300 µg/mg were specified to have microalbuminuria.

Data analysis

Descriptive and inferential statistics were applied for the data analysis in Statistical Package for the Social Sciences (SPSS) version 25 (Armonk, NY: IBM Corp.). By the implementation of descriptive statistics, qualitative data were expressed in terms of frequency and percentage, whereas quantitative data were described in terms of means ± standard deviation (SD). A chi-square test was practiced to estimate the association of VD status with gender, age group, and albuminuria status. Independent-sample t-test was used to compare the means of UACR between the diabetic patients with VD deficiency and non-VD deficiency groups. Pearson correlation was applied to determine the correlation between the VD level and UACR. p-Value less than 0.05 was set as statistically significant.

## Results

Of the total of 110 diabetic patients, 60% (n=66) were females, whereas 40% (n=44) were males. The means for the study population for different variables like age, VD, and UACR were 48.50 years with an SD of ±15.67 years, 28.16 with an SD of ±15.34 ng/mL, and 29.69 with an SD of ±87.22 µg/mg, respectively. The overall incidences of VD deficiency and microalbuminuria in the study population were 43.64% (n=48) and 28.20% (n=31), respectively.

Table 1 elaborates that VD deficiency incidence was higher among the older age group, females, and in patients with microalbuminuria in contrast to the younger age group, males, and in patients with normo-albuminuria. Likewise, it also shows that the association of VD level with age group, gender, and albuminuria status was statistically significant. 

**Table 1:** Demographic and serum vitamin D status of the study population along with cross-tabulation and chi-square test analysis of study variables

Variables			Cross-tabulation and chi-square test		
Total count=110		N (%)	Serum vitamin D levels		p-Value
			Vitamin D deficiency 48 (43.64%)	Non-Vitamin D deficiency 62 (56.36%)	
Age group (years)	30 to 45	54 (49.10%)	20 (41.66%)	34 (54.84%)	0.002
	Above 45 to 65	56 (50.90%)	28 (58.34%)	28 (45.16%)	
Gender	Male	44 (40%)	18 (37.50%)	26 (41.93%)	0.008
	Female	66 (60%)	30 (62.50%)	36 (58.07%)	
Albuminuria status	Normo-albuminuria	79 (71.80%)	19 (39.58%)	60 (96.77%)	0.004
	Microalbuminuria	31 (28.20%)	29 (60.42%)	02 (3.23%)	

Table 2 indicates that the UACR mean was higher among the VD deficiency group in comparison to the non-VD deficiency group and this difference in means between these groups was statistically significant.

**Table 2:** Comparison of means of UACR between the vitamin D deficiency group and non-vitamin D deficiency group and independent t-test analysis UACR, urine spot for albumin-to-creatinine ratio.

Variable	Mean±SD	Serum vitamin D status		Independent-samples t-test
		Vitamin D deficiency	Non-vitamin D deficiency	p-Value
UACR (µg/mg)	29.69±87.22	56.43±60.12	14.93±32.69	0.001

Table 3 shows that with the increase in UACR value the serum VD level goes down and the correlation between the UACR/microalbuminuria and serum VD level was negative and statistically significant.

**Table 3:** Pearson correlation analysis between serum vitamin D level and UACR UACR, urine spot for albumin-to-creatinine ratio.

Variable	Correlation coefficient (r)	p-Value
UACR	-0.60	0.002

## Discussion

This study has highlighted the incidence of VD deficiency and the association of VD deficiency with age group, gender, and microalbuminuria among the patients with T2DM in a tertiary care hospital in Rawalpindi, Pakistan.

In the present study, the incidence of VD deficiency in patients with T2DM was quite high (43.64%). Another study in Pakistan has recorded a little lower incidence of VD deficiency in patients with T2DM (41.50%) [10]. Various studies around the globe have reported a prevalence of VD deficiency in patients with T2DM that ranges from 29.50% to 91% [6,11]. The large range of the prevalence of VD deficiency in T2DM patients could be due to several factors like geographical, racial, dietary, and most importantly the stage of T2DM in patients. Many researches have shown that VD plays an important role in the pathogenesis of T2DM. VD deficiency leads to a reduction in insulin production and stops the conversion of inactive form proinsulin into insulin active form as well. Furthermore, VD deficiency also causes insulin insensitivity [4,12]. Whereas, adequate replacement of VD not only improves the blood glucose level via controlling the insulin signaling but also protects against cardiovascular diseases, autoimmune diseases, infectious diseases, and musculoskeletal disorders by regulating lipogenesis and inflammation [4,6,11].

The association between serum VD levels and age group was statistically significant. VD deficiency incidence was higher in T2DM patients in the higher age group (58.34%) in contrast to T2DM patients in the lower age group (41.66%). This finding of the present study was also endorsed by another study that was also conducted in patients with T2DM [13], whereas an American study noted a conflicting finding and showed a higher incidence of VD deficiency in the younger population with T2DM [14]. Another important observation of this study regarding the relationship between serum VD levels and gender showed that the incidence of VD deficiency was higher in women (62.50%) than men (37.50%). Moreover, the association between serum VD levels and gender was also statistically significant in the current study population. The higher prevalence of VD deficiency in women and a significant association of gender with serum VD level were also noted in a study that was carried out in Sudan [15].

Serum VD level and albuminuria status were also associated significantly. Serum VD deficiency was greater in T2DM patients with microalbuminuria (60.42%) as compared to T2DM patients with normo-albuminuria (39.58%). A Turkish study supported this finding of the present study and recorded a higher incidence of VD deficiency in T2DM patients with microalbuminuria. Furthermore, this study also found a statistically significant relationship between the serum VD level and the albuminuria status like the present study [4]. Another study that was performed in Qatar noted a higher incidence of VD deficiency in T2DM patients with microalbuminuria [6].

It was also noted that the mean value of UACR was higher in the VD deficiency group in contrast to that in the non-VD deficiency group. Likewise, we also observed a statistically significant and inverse correlation between the VD level and microalbuminuria. A Sudanese study also agrees with the findings of the present study [6]. It has been recorded in different studies that VD deficiency in patients with diabetes mellitus raises the risk of macrovascular and microvascular complications. Moreover, the increase in the frequency of VD deficiency in patients with microalbuminuria suggests that VD could have a role in the development of diabetic nephropathy as studies have recommended that replacement of VD in T2DM patients brings a decline in albuminuria in patients with chronic kidney disease [16,17]. In the presence of a significant association between VD deficiency and microalbuminuria, we recommend that serum VD level should be monitored and replaced in patients with T2DM intermittently and this would prevent the progression of diabetic nephropathy.

The limitation of this study is linked to its cross-sectional design because of which the causal correlation between the serum VD level and albuminuria could not be explained. Therefore, further studies with different study designs are needed that could elaborate on the temporal association between the serum VD level and albuminuria.

## Conclusions

The current study population had a high incidence of VD deficiency (43.64%). VD deficiency had a statistically significant association with age group, gender, and albuminuria status in the study population. T2DM patients with higher age, female gender, and microalbuminuria had a higher incidence of VD deficiency relative to T2DM patients with lower age, male gender, and normo-albuminuria. Furthermore, a significant and negative correlation was also observed between the serum VD level and microalbuminuria, which means that with the decrease in serum VD level the incidence of microalbuminuria goes up in patients with T2DM. This study recommends that the VD level should be monitored in patients with T2DM for the prevention of further complications by prompt replacement of VD.

## Appendices

**Table 4:** Self-designed and interview-based proforma

Research proforma	Incidence of vitamin D deficiency and its association with microalbuminuria in patients with type 2 diabetes mellitus			
Question number	Research questions	Options; Write and mark the option		
1	What is your age?	…….. in years	30 to 45 years	Above 45 to below 65 years
2	What is your gender?	Male	Female	
3	What is the serum vitamin D level?	…… ng/mL		
4	What is your group based on your serum vitamin D level?	Vitamin D deficiency group (less than 20 ng/mL)	Non-vitamin D deficiency group (above 20 ng/mL)	
5	What is your urine albumin-to-creatinine ratio?	……µg/mg		
6	What is your albuminuria status based on your urine albumin-to-creatinine ratio?	Normo-albuminuria (less than 30 µg/mg)	Microalbuminuria (between 30 and 300 µg/mg)	
